# Increase of the Antarctic Sea Ice Extent is highly significant only in the Ross Sea

**DOI:** 10.1038/srep41096

**Published:** 2017-01-24

**Authors:** Naiming Yuan, Minghu Ding, Josef Ludescher, Armin Bunde

**Affiliations:** 1 CAS Key Laboratory of Regional Climate-Environment for Temperate East Asia, Institute of Atmospheric Physics, Chinese Academy of Sciences, Beijing, 100029, China; 2 Department of Geography, Climatology, Climate Dynamics and Climate Change, Justus Liebig University Giessen, 35390 Giessen, Germany; 3 Chinese Academy of Meteorological Science, Beijing, 100081, China; 4 Institute for Theoretical Physics, Justus Liebig University Giessen, Heinrich-Buff-Ring 16, D-35392 Giessen, Germany

## Abstract

In the context of global warming, the question of why Antarctic sea ice extent (SIE) has increased is one of the most fundamental unsolved mysteries. Although many mechanisms have been proposed, it is still unclear whether the increasing trend is anthropogenically originated or only caused by internal natural variability. In this study, we employ a new method where the underlying natural persistence in the Antarctic SIE can be correctly accounted for. We find that the Antarctic SIE is not simply short-term persistent as assumed in the standard significance analysis, but actually characterized by a combination of both short- and long-term persistence. By generating surrogate data with the same persistence properties, the SIE trends over Antarctica (as well as five sub-regions) are evaluated using Monte-Carlo simulations. It is found that the SIE trends over most sub-regions of Antarctica are not statistically significant. Only the SIE over Ross Sea has experienced a highly significant increasing trend (*p* = 0.008) which cannot be explained by natural variability. Influenced by the positive SIE trend over Ross Sea, the SIE over the entire Antarctica also increased over the past decades, but the trend is only at the edge of being significant (*p* = 0.034).

Polar sea ice, as an important component of the climate system, can affect the earth system in many ways. It hinders exchanges between ocean and atmosphere, reflects solar radiation back to space, and even changes the habitat and species of plants and animals in the polar region^1^,^2^. In the context of global warming, it is of great importance to monitor the variations of polar sea ice and further study the potential effects of polar sea ice changes^3^,^4^. In the Arctic, a significant decreasing trend of the sea ice extent (SIE) has been well recognized, which is due to the Arctic warming^5^,^6^. However, unlike the Arctic SIE, recent satellite observations surprisingly reveal a positive trend in the Antarctic SIE^7^,^8^, which confounds the most trusted climate models in the world, including the Coupled Model Intercomparison Project Phase 5 (CMIP5) models^9^,^10^. Therefore, understanding the unexpected increasing trend of the Antarctic SIE has become a research hotspot in the past decades.

Among the natural explanations are the effects of El Niño events, the Southern Annular Mode (SAM), wind patterns as well as geology, etc.^11^,^12^,^13^,^14^. Among the anthropogenic explanations are, apart from global warming, the ozone depletion, which may both deepen the Amundsen Sea Low (ASL), strengthen the southernly winds over the Ross Sea, and finally lead to an increasing SIE trend over this area^15^,^16^,^17^,^18^. The ozone hole also may induce a positive SAM trend, which in turn may lead to an increasing trend of the Antarctic SIE. However, coupled atmosphere-ocean-sea ice models suggest that the ozone depletion may lead to a negative trend of the Antarctic SIE^19^,^20^,^21^.

Up to now, the reasons why the SIE shows a positive trend are still in debate. But beyond the reasons for a positive trend, a more important question is, whether the trend is originated from anthropogenic forcings, or within the bounds of natural variability. All previous calculations of the trend significance concluded that the positive trend of the entire Antarctic SIE over the past 30 years is statistically highly significant^8^, which indicates the positive trend is unexplainable using natural variability. However, from model simulations, previous studies argued that it may be difficult to attribute the positive SIE trend over Antarctica to anthropogenic forcings, since the distribution of the simulated natural trend may be wide enough to cover the observed Antarctic sea ice trend^15^,^22^,^23^,^24^. Therefore, it is still an open question whether the Antarctic SIE trend is significant or not. To make a better estimation of the trend significance, it is crucial to know its accurate natural variability including its persistence properties.

Figure 1 shows the time evolution of the Antarctic SIE anomalies between November 1978 and December 2013 in the five Antarctic subregions^25^. The SIE anomalies were calculated by subtracting the annual cycle (see the “Data” section). To show the temporal variation more clearly, we made a moving average over 30 months (see the green curves). In the figure, one can see clearly a pronounced mountain-valley structure which represents the variability caused by the natural persistence and anthropogenic forcings. In the previous calculations of the trend significance, it has been assumed that the natural part of the Antarctic SIE exhibits only short-term persistence, which can be modeled by an auto-regression process of first order (AR(1)). In this case, the autocorrelation function *C(s*) decays exponentially with time lag *s* as 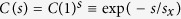, and the statistical significance of the Antarctic SIE trend can be determined from the classic method developed by^26^ (see “Method” section). However, it has been well recognized that another type of persistence, long-term persistence, is more relevant in climate^27^ (see “Method” section). Different from short-term persistence, long-term persistence indicates long lasting influences from the past histories and its autocorrelation function *C(s*) decays asymptotically as *C(s*) ~ (1 − *γ)s*^−*γ*^ (*s* > 0, see “Method” section). In general, both short- and long-term persistence can contribute to the observed natural variability, and a correct estimation of the underlying persistence is a prerequisite for a proper estimation of the trend significance^28^,^29^,^30^,^31^,^32^. So far, this kind of analysis has not been done for the Antarctic SIE.

In this study, we apply Detrended Fluctuation Analysis of second order^33^ (DFA2, see “Method” section) to the monthly SIE anomalies to estimate the persistence of the SIE. We show explicitely that the persistence can be modeled by a superposition of short- and long-term persistent processes. By using extensive Monte-Carlo simulations, we determine the desired distribution of natural trends, which finally allows us to determine how significant the SIE trends in the Antarctic regions are.

Figure 2 shows the DFA2 fluctuation function *F(s*) of the SIE for the 5 regions and the entire Antarctica (black dots). In the double-logarithmic presentation one can see that the fluctuation functions *F(s*) show, for all SIE data, a crossover at *s* between 18 and 20 months. The steeper slopes below the crossovers indicate short-term persistence. Above the crossover, *F(s*) increases by a power law, *F(s*) ~ *s*^*h*^, with the Hurst exponent *h* between 0.6 and 0.8, indicating long-term persistence on large time scales. Accordingly, the case of pure short-term persistence where *h* = 0.5 can be excluded. The Hurst exponent characterizes the strength of the long-term persistence: the larger *h* the higher the persistence. The figure suggests that the natural variability of the Antarctic SIE can be described by a *superposition* of short- and long-term persistence processes, which can be modeled by (see “Method” section)


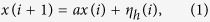


where *a* is the AR(1) parameter and *η*_*h*_(*i*) is long-term persistent noise. We like to note that equation (1) reduces to the standard AR(1) model when *η*_*h*_(*i*) is substituted by Gaussian white noise. On the other hand, for *a* = 0, it reduces to the standard model of long-term persistence.

It is not surprising that the Antarctic SIE shows, on large time scales, long-term persistence, since also the surrounding sea and air temperatures are known to be long-term persistent^30^,^31^,^32^. A possible origin of the long-term persistence of the Antarctic SIE is the internal dynamics of sea ice. As discussed in refs 34 and 35, processes including the Southern Annular Mode (SAM), El Niño-Southern Oscillation (ENSO), high-frequency weather forcings, as well as the South Pacific Intrinsic Mode (SPIM) can drive the multi-scale sea ice variability, which may further induce the arise of long-term persistence.

Using equation (1), we have determined the appropriate parameters *a* and *h* for all Antarctic regions by best fits of the fluctuation functions *F(s*). The values of *a* and *h* are shown in Fig. 2. For all five regions, the estimated AR(1) parameters are similar ranging from *a* = 0.5 to 0.55, indicating the short-term persistence of SIE over different Antarctic sub-regions may have similar dynamical sources. Regarding the long-term persistence, the parameter *h* varies from 0.6 in the “Ross Sea” and “Pacific Ocean”, to 0.8 in the “Indian Ocean”, which may imply different mechanisms in producing the long-term persistence. As discussed in refs 34 and 35, the different roles of SAM, ENSO, as well as the high-frequency weather forcings in driving sea-ice variability over different regions, may contribute to the different *h* values. Using these values of *a* and *h*, as the figure shows, the DFA2 results of the simulated data agree well with those of the observed SIE data.

For each SIE data set, we then used equation (1) with the proper *a* and *h* values, to generate more than 100,000 artificial data sets with the same length *L* = 422 which by definition mimic the natural variability of the considered SIE data set. By determining the relative trend *x* in each artificial record, we then arrive, for each of the 6 SIE data sets, at the desired probability density function *P(x*) and the related statistical significance *S(x*) of the observed trend, as well as the *p*-values *p(x*) = 1 − *S(x*) (see “Method” section).

Figure 3 shows the *p*-values for the artificial data sets (green line) simulating the natural variability of (a) the Weddell Sea, (b) Bellingshausen and Amundsen Seas, (c) Ross Sea, (d) Indian Ocean, (e) Pacific Ocean, and (f) entire Antarctica. In order to reveal how sensitively the statistical significance depends on the assumption of the underlying persistence of each record, we show also *p(x*) for (i) white noise and (ii) the corresponding AR(1) process. For both white noise and AR(1) processes, *p(x*) can be obtained straightforwardly from equation (7) in the Methods Section. In addition, (iii) we have determined *p(x*) for long-term persistent data with the same *h* values and the same length *L* = 422 as the original SIE data. For details of the calculation we refer to ref. 29.

The vertical dashed line in each panel represents the relative trend *x* of the SIE in each of the 6 Antarctic regions. The intersection of this vertical line with *p(x*) yields the desired *p* value (horizontal dashed line). The figure shows the dependence of *p* on the considered statistical model for the natural variability. Under the assumption of white noise (black line), the SIE trend in all regions is highly significant, with *p*-values well below 0.01. Under the standard assumption of an AR(1) process (red line), the SIE trends in Ross Sea, Indian Ocean, and Antarctica are highly significant, with *p* values well below 0.01. For the other 3 regions, the trends are not significant since *p* is above 0.05. Under the assumption of a purely long-term persistent process (blue line), the trends in the Ross Sea and entire Antarctica are highly significant, with *p* values below 0.01. For the appropriate assumption of a combined short- and long-term persistent process (equation (1)), we find that the trend in the SIE is not significant in 4 of the 5 Antarctic subregions where the *p* values are well above 0.2. Only in the Ross Sea the trend is highly significant (*p* = 0.008). This result suggests that only the positive trend of the SIE in the Ross Sea cannot be explained by its natural variability, whereas the trends in the other 4 subregions are well within the bounds of natural variability. In the entire Antarctica, the trend is only slightly significant, with *p* = 0.034 only little below the significance threshold 0.05.

In addition to the significance, we have also determined the maximum and minimum external trend (see equation (5) and (6) in the “Method” section) for the significance levels *α* = 0.05 and 0.01. Figure 4 summarizes all results for the SIE in the 6 Antarctic regions, (a) for the standard assumption of an AR(1) process, and (b) for the combined AR(1) and long-term persistent process. The numbers shown are the *p*-values. The upper and lower bounds of the boxes represent the maximum and minimum external trend for the significance level *α* = 0.05, while the error bars stand for the maximum and minimum external trend for *α* = 0.01. The figure again shows that the assumption of an AR(1) process strongly underestimates the uncertainties in the Antarctic SIE and overestimates the significance. A remarkable difference between (a) and (b) occurs in the Indian Ocean. The conventional AR(1) treatment yields that the increase of the SIE is highly significant, with *p* = 0.008. On the other hand, when the considerable long-term persistence (*h* = 0.8) of the SIE in the Indian Ocean is taken into account, *p* becomes 0.26, which is well above the significance level 0.05.

## Discussion and Conclusion

In this study, we presented evidence that the temporal evolution of the Antarctic SIE is characterized by a superposition of both, short-term persistent and long-term persistent processes. By combining both processes (see equation (1)), we succeeded in obtaining a realistic statistical model for the natural variability of the Antarctic SIE. The model allowed us to determine, by Monte-Carlo simulations, the natural distribution of trends and thus the statistical significance of the trends in the Antarctic SIE. We find that already in the considered small time window of 35 years the SIE trend over Ross Sea is highly statistically significant with *p* = 0.008, while the trends of the other four sub-regions are well within the bounds of natural variability, with *p*-values well above 0.2. Accordingly, only the trend in the Ross Sea SIE cannot be explained by natural variability alone and external forcings must contribute to this trend. For the other four sub-regions, the SIE trends are not statistically significant, either because there is no anthropogenic trend or because the considered time period is not long enough to distinguish a deterministic anthropogenic trend from the natural fluctuations of the records. The SIE over the entire Antarctica increased over the past decades, but the trend is only at the edge of being significant (*p* = 0.034).

Our work, from a new perspective, resolved the discrepancy reported by the previous studies. On one hand, statistical models based on short-term persistence only, suggest that the Antarctic SIE trends are highly significant and cannot be explained by natural variability^8^. On the other hand, dynamical model simulations suggested that the observed SIE trends may be not strong enough to exceed the bounds of the natural variabilities of SIE^22^,^24^. But it is not sure how trustworthy the simulations are, since they are unable to reproduce the observed increase in the Antarctic SIE. Here we have shown that the main reason for the great discrepancy between the previous statistical analysis and the dynamical model simulations is the inappropriate assumption of short-term persistence in the statistical model. Possible defects in the ability of the dynamical models in simulating the Antarctic SIE^9^,^10^ do not play a major role here.

Based on our analysis, Ross Sea is the only subregion that has been affected strongly by the anthropogenic forcings. The mechanism may be as follows^15^,^16^,^17^,^18^,^36^: Both global warming and ozone depletion may deepen the Amundsen Sea Low (ASL), weaken the westerlies near the Ross Sea and strengthen the southerly cold winds from the continent. This way, the sea ice in the Ross Sea may become more isolated and increase due to the southerly cold winds.

The fact that the CMIP5 models are not able to reproduce the increase of the SIE in Antarctica, points to a remarkable deficiency of the models, even though the models tried to implement the effects of the ozone depletion^19^,^20^,^21^. Since the Ross Sea is the main driver of this increase, we suggest that the models concentrate on the Ross Sea in order to see what went wrong and how to improve the performance.

## Data and Methods

### Data

**Figure 1:**
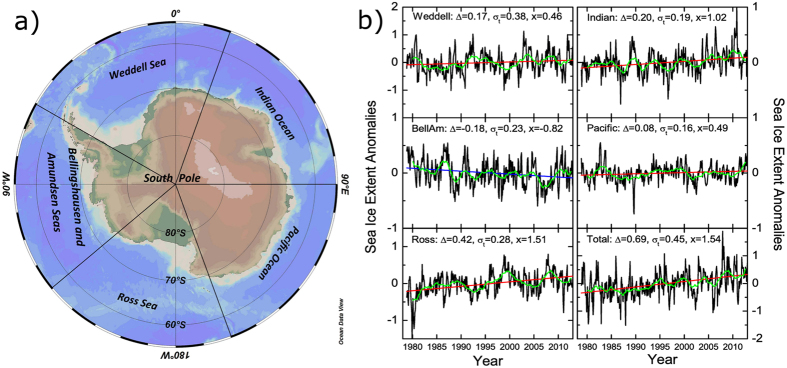
Identification of the five sub-regions and their corresponding Sea Ice Extent anomalies. (**a**) Shows the five sub-regions which are studied in this work. They are: Weddell Sea (60W-20E), Indian Ocean (20-90E), western Pacific Ocean (90-160E), Ross Sea (160E-130W), and the combined Bellingshausen and Amundsen Seas (130-60 W). (**b**) Shows temporal evolutions of the SIE anomalies for the five sub-regions, as well as the total Antarctica. SIE anomalies are calculated by subtracting the annual cycle (see “Data” section). To show better show the temporal variation, moving average over 30 months are calculated (green curves). Linear regression lines are fitted for each time series, and the values of three quantity of interest, Δ (magnitude of the trend), *σ*_*t*_ (fluctuations around the trend), *x* (relative trend), are shown in each sub-figure. As one can see, only one sub-region (BellAm) shows a negative trend of the SIE. For all the other four sub-regions and the total Antarctic, there are positive SIE trends. a) was generated using the software “Ocean Data View (ODV)” version 4.7.7^40^, for details please refer to http://odv.awi.de/.

In this study, the trends of the SIE over Antarctica are investigated using the monthly sea ice data set from the US National Snow and Ice Data Center (NSIDC) (Data contributors: Stroeve, J. and Meier, W., http://nsidc.org/data/nsidc-0192). Monthly mean sea ice concentrations were used to derive the extents, which is derived from passive microwave measurements at a grid cell size of 25 × 25 km from Nimbus-7 SMMR; DMSP-F8, -F11, and -F13 SSM/I; and from DMSP-F17 SSMIS. NASA team Algorithms is applied in computing the total SIE. Total SIE is derived by summing the number of pixels with at least 15 percent ice concentration multiplied by the area per pixel. In this way, the entire area of any pixel with at least 15 percent ice concentration is considered to contribute to the total SIE. According to ref. 37, it is suggested that using monthly mean sea ice concentrations may provide a more accurate monthly ice extent since the effects of storms on the ice edge are less pronounced in the monthly mean sea ice data than in the daily sea ice data. Besides the SIE of the entire Antarctic, the SIE over the sector of Bellingshausen and Amundsen Sea, Southern Pacific Ocean, Southern Indian Ocean, Weddell Sea and Ross Sea is also calculated, with the same protocol of Zwall *et al*.^25^ and Turner *et al*.^15^. SIE data employed in this study ranges from November 1978 to December 2013 (422 months in total). Before analysis, the SIE anomalies are calculated by subtracting the annual cycle, which is defined as the averages of each calendar month (see Fig. 1b).

## Methods

### Short- and long-term persistence

In records with short-term persistence, the autocorrelation function *C(s*) of the (detrended) anomalies decays exponentially as 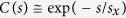. In records with long-term persistence, *C(s*) decays algebraically as *C(s*) ≅ (1 − *γ)s*^−*γ*^, *s* > 0, 0 < γ < 1. In some cases like the Antarctic SIE, the persistence of the record is characterized by the superposition of short- and long-term persistence. All the above three cases can be modeled as following,


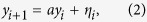


where *a* is the AR(1) parameter and *η*_*i*_ is long-term persistent noise. Basically, this is a generalization of an auto-regression process of first order (AR(1)). By setting *η*_*i*_ as white noise, equation (2) will be degenerated as AR(1) process, which is useful for the modeling of short-term persistent records. In this case, the persistent time 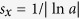. By setting the AR(1) parameter as *a* = 0, equation (2) will be able to model purely long-term persistent data. In this case, the persistent time *s*_*x*_ diverges. While by using appropriate AR(1) parameter *a* and long-term persistent data *η*_*i*_, equation (2) can be used to simulate the natural variability of data with combined short- and long-term persistence. Numerically, a long-term persistent data set {*η*_1_, *η*_2_ … *η*_*N*_} can be generated by the Fourier Filtering Technique^38^. The data sets are characterized by the correlation exponent *γ* or the Hurst exponent *h* (see below). Then, by choosing *y*_1_ randomly from a Gaussian distribution, the values for *y*_2_, *y*_3_ … *y*_*N*_ can be calculated recursively from (2).

### Detrended Fluctuation Analysis

Since *C(s*) shows strong finite size effects, one usually considers the Detrended Fluctuation Analysis 2 (DFA2) to detect the underlying persistence in the record of interest. In DFA2, one first divides the record {*y*_*i*_}, *i*  = 1, …, *L*, into non-overlapping windows *μ* of lengths *s*. Then one focuses, in each segment *μ*, on the cumulated sum *Y*_*i*_ of the data and determines the variance 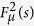 of the *Y*_*i*_ around the best polynomial fit of order 2. After averaging 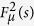 over all segments *μ* and taking the square root, one arrives at the desired fluctuation function *F(s*)^33^. *F(s*) reflects the different kinds of persistence in the following way:

- Without any persistence, for simple white noise, *F(s*) increases as *s*^1/2^.
- In short-term persistent records with a cross-over time *s*_*x*_, *F(s*) shows a cross-over. For *s* well above *s*_*x*_, *F(s*) increases as *s*^1/2^ as for white noise, while below *s*_*x*_, *F* increases stronger than *s*^1/2^. For very large *s*_*x*_, *F* increases as *s*^3/2^ for *s* below *s*_*x*_.
- In purely long-term persistent records, *F(s*) increases as

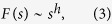


where the exponent *h* can be associated with the Hurst exponent and is related to the correlation exponent *γ* by *h* = 1 − *γ*/2. For stationary records, the dimension-less Hurst exponent *h* is between 0.5 and 1; *h* characterizes the strength of the long-term persistence. For increasing *h*, the strength of the long-term persistence increases and the mountain valley structure caused by the persistence becomes more pronounced.

When both short- and long-term persistence are present, *F(s*) shows a crossover similar to that one of short-term persistent records. For *s* below *s*_*x*_, *F* increases as in short-term persistent records, while for *s* well above *s*_*x*_, *F* increases as for purely long-term persistent records.

### Significance of trends

We consider the record {*y*_*i*_} with length *L*. From the regression line *r*_*i*_ = *ai* + *b*, we obtain the magnitude of the trend Δ = *a(L* − 1) and the fluctuations around the trend, characterized by the standard deviation 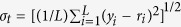. The relevant quantity we are interested in is the *relative trend*


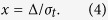


For assessing if an observed trend in a data set may be due to its natural persistence or not, one needs to know the probability *P(x, L)dx* that in model records with the same persistence properties as the considered data set, a relative trend between *x* and *x* + *dx* occurs. From *P* one obtains the statistical significance *S* of the trend *x*, 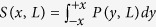. The deviation of *S* from 1 is called *p*-value.

If *p(x, L*) is below a certain significance level *α* (usually *α* is 0.05 or 0.01), one usually assumes that the considered trend cannot be fully explained by the natural variability of the record. The relation *p(x*_*α*_;*L*) = 1 − *S(x*_*α*_;*L*) = *α* defines the upper and lower limits ±*x*_*α*_ of the considered significance interval (also called confidence interval). By the above assumption, relative trends *x* between −*x*_*α*_(*L*) and *x*_*α*_(*L*) can be regarded as natural. If *x* is above *x*_*α*_, the part *x* − *x*_*α*_ cannot be explained by the natural variability of the record and thus can be regarded as minimum external relative trend,


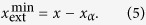


Similarly, the external relative trend cannot exceed


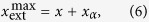


which thus represents the maximum external relative trend. According to (5) and (6), ±*x*_*α*_(*L*) can be regarded as error bars for an external relative trend in a record of length *L*.

For Gaussian white noise, *S* is given by


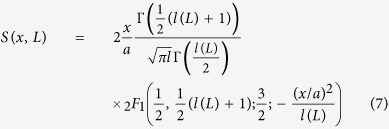


with *l(L*) = *L* − 2 and *a* = (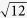
(*L* − 1)/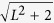)/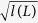 ≅ 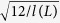. _2_*F*_1_ is the hypergeometric function and Γ denotes the Γ-function.

For short-term persistent records described by an autoregressive process of first order (AR(1)), Eq. (7) remains the same, only *l(L*) has to be substituted by *L*(1 − *C*(1))/(1 + *C*(1)), where *C*(1) is the auto-correlation with lag of 1 (months).

For long-term persistent records characterized by a Hurst exponent *h, S(x, L*) has the same functional form as in Eq. (7). The parameters *l* and *a* depend on *L* and *h* and have been tabulated in ref. 39.

## Additional Information

**How to cite this article:** Yuan, N. *et al*. Increase of the Antarctic Sea Ice Extent is highly significant only in the Ross Sea. *Sci. Rep.* **7**, 41096; doi: 10.1038/srep41096 (2017).

**Publisher's note:** Springer Nature remains neutral with regard to jurisdictional claims in published maps and institutional affiliations.

## Figures and Tables

**Figure 2:**
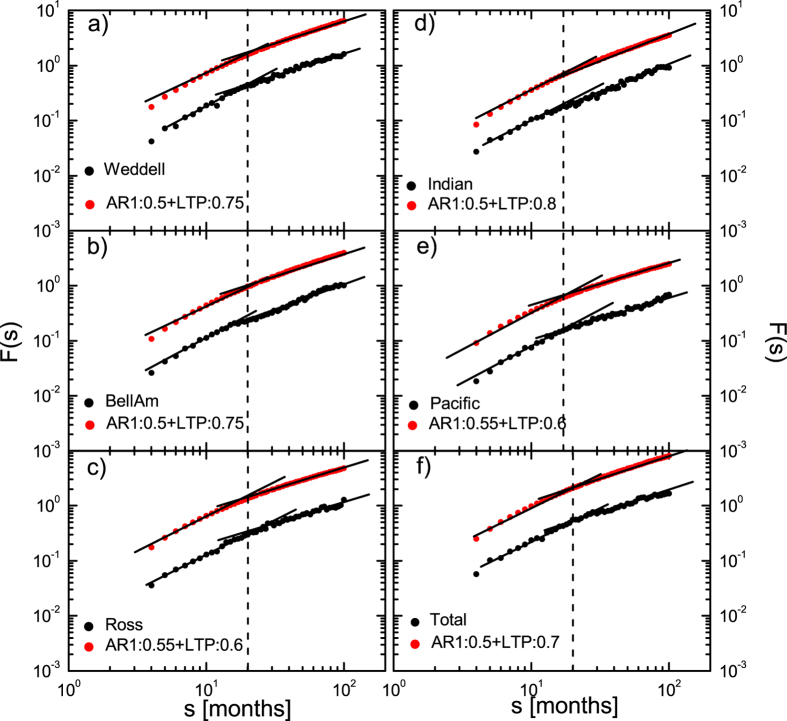
DFA2 results for both SIE data and surrogate data. (**a–e**) Are the DFA2 results for the five sub-regions, while (**f**) shows the DFA2 results for the entire Antarctic. *F(s*) is the fluctuation function calculated from DFA (see equation (3) in the “Method” section). The black dots represent the results obtained from the instrumental SIE data, and the red dots represents the results from computer generated surrogate data. To better show the results, the red dots in each sub-figure are shifted upwards by distance of *log*_10_(4). As one can see, there are crossovers in the DFA2 fluctuation functions in all regions, with crossover times around 18–20 months (see the vertical dashed line), indicating a mixture of both short and long-term persistence. By using appropriate AR(1) parameters and Hurst exponents which are marked in each panel, the surrogate data show the same persistent properties as the SIE data.

**Figure 3:**
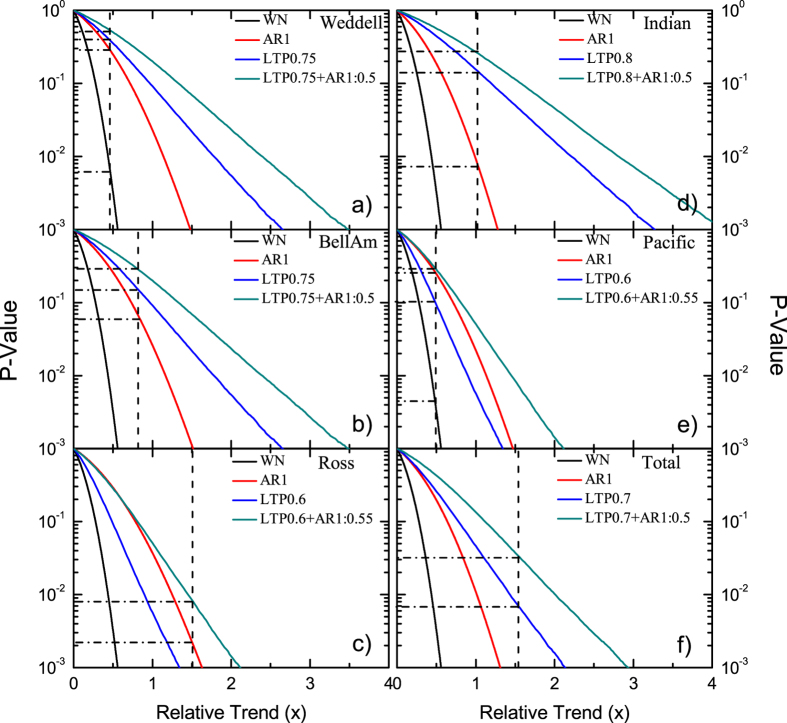
*p*-values for trend evaluation. (**a–e**) Are the results for the five sub-regions, while (**f**) shows the results for the entire Antarctic. The four curves in each panel represent the *p*-values vs the absolute value of the dimensionless relative trend *x*, for four different assumptions: (i) white noise (WN, black curves), (ii) AR(1) (AR1, red curves), (iii) long-term persistent process (LTP, blue curves), and (iv) combined AR(1) and long-term persistent processes (LTP + AR1, cyan curves). The vertical dashed line in each panel shows the relative trend *x* of each SIE data set. The horizontal dashed line points to the corresponding *p*-values. The figure shows that for the most realistic scenario (iv), only the positive trends in the Ross Sea and in the entire Antarctic are statistically significant.

**Figure 4:**
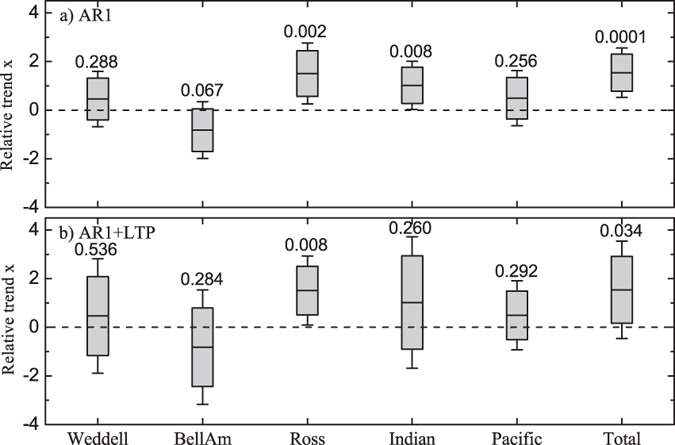
Maximum and minimum external trends for the Antarctic regions. (**a**) For the standard assumption of an AR(1) process, (**b**) for the combined AR(1) and long-term persistent process. The upper and lower bounds of the boxes show the maximum and minimum external trends for the significance level of *p* = 0.05, while the error bars represent the maximum and minimum external trend for the significance level of *p* = 0.01. The number over each box is the corresponding *p*-value.
